# Blood as the mirror and modulator of aging: mechanistic insights and rejuvenation strategies

**DOI:** 10.1038/s12276-026-01688-1

**Published:** 2026-04-17

**Authors:** Eunhwan Kim, Jae Sook Kang, Yong Ryoul Yang

**Affiliations:** 1 Aging Convergence Research Center, Korea Research Institute of Bioscience and Biotechnology (KRIBB), Daejeon, Republic of Korea; 2 Department of Bimolecular Science, KRIBB School of Bioscience, Korea University of Science and Technology (UST), Daejeon, Republic of Korea

**Keywords:** Ageing, Translational research

## Abstract

Aging arises not only from intrinsic cellular decline but also from systemic alterations in circulating factors that govern tissue maintenance and regeneration. Recent multi-omics advances — including plasma proteomics, metabolomics, and single-cell immunomics — highlight blood as both a mirror and a modulator of organismal aging. Circulating proteins and metabolites reflect not only chronological and biological age but also organ-specific aging trajectories, serving as robust predictors of healthspan, longevity, and disease risk. Beyond their diagnostic value, blood-borne components actively dictate the tempo of aging by shaping immune remodeling, metabolic homeostasis, and interorgan communication. Youthful circulation, defined as the blood-borne systemic environment of young individuals, promotes tissue homeostasis and regeneration and, when experimentally transferred via heterochronic parabiosis or young plasma transfer, induces transcriptomic, metabolic, and epigenetic rejuvenation across multiple tissues. Specific fractions — such as small extracellular vesicles, plasma proteins, and metabolites — restore mitochondrial function, suppress inflammation, and extend lifespan in animal models. Conversely, reducing pro-aging factors through plasma dilution or therapeutic plasma exchange mitigates age-associated decline and shows translational promise in neurodegenerative disease. Collectively, these insights position blood as a central regulatory axis of aging. In this Review, we synthesize current mechanistic and translational evidence on blood-borne aging regulators to outline a molecular framework for rejuvenation biology and future therapeutic development.

## Blood factors shape aging and regeneration pathways

Aging is a complex process influenced by changes in our blood that affect how quickly we age. Scientists have shown that blood contains important molecules and cellular components — including proteins, metabolites, and immune cells — that can either accelerate or slow aging. Tools such as the ‘proteomic aging clock’ predict age and disease risk based on blood protein profiles, whereas emerging multi-omics approaches integrate metabolomic and immunomic data. Large-scale analyses of circulating factors reveal how these components change with age and identify markers of organ-specific aging. Certain blood-borne molecules can predict diseases such as heart disease and Alzheimer disease. These findings demonstrate that aging does not occur uniformly across tissues. Overall, studying diverse blood components provides valuable insight into aging biology and offers opportunities to develop strategies that promote healthier aging and improve long-term health.

This summary was initially drafted using artificial intelligence, then revised and fact-checked by the author.

## Introduction

Aging is a multifaceted biological process driven by the accumulation of molecular damage, epigenetic alterations, and impaired tissue communication^1^. Although the cellular hallmarks of aging have been widely characterized, increasing evidence indicates that systemic factors circulating in the blood are equally critical in determining the rate and quality of aging. The concept that blood can influence organismal aging dates back to classic parabiosis experiments, which revealed that exposure of old organisms to young circulation can restore regenerative capacity and organ function^2^. These pioneering observations have redefined aging as a reversible and systemically regulated process rather than a purely intrinsic decline.

With the advent of high-resolution proteomics, metabolomics, and single-cell technologies, the blood has emerged as a molecular map of aging. Plasma proteins and metabolites are emerging as promising biomarkers that may reflect both chronological and biological aspects of aging. Proteomic aging clocks derived from large population cohorts accurately predict not only age but also future disease risk and mortality^3^. Moreover, tissue-specific and organ-specific plasma signatures reveal that aging progresses along distinct trajectories across organs, highlighting the brain and immune system as central regulators of healthspan and longevity^4^. In parallel, single-cell immune profiling has unveiled age-associated shifts in blood immune cell composition, including loss of naive lymphocytes, expansion of exhausted T cells, and chronic low-grade inflammation known as inflammaging^5,6^. These immune alterations are not merely consequences but active drivers of systemic aging, propagating senescence and metabolic dysfunction across tissues.

Accumulating evidence indicates that the systemic circulation contains both rejuvenating and pro-aging components that exert opposing effects on tissue homeostasis. Rejuvenating factors, enriched in young plasma or restored through circulatory interventions, promote enhanced stem-cell activity, mitochondrial function, and tissue repair across multiple organs. By contrast, pro-aging circulating factors — including inflammatory cytokines, chemokines, and damage-associated molecular patterns — are linked to impaired regeneration, immune dysfunction, and organ-specific decline, particularly in the brain, muscle, and immune system. Consequently, the balance between these antagonistic circulatory signals, rather than individual factors alone, may critically modulate systemic aging trajectories.

Consistent with this framework, experimental evidence increasingly supports that alterations in circulating factors and immune cell composition have a causal role in regulating the aging process. Experimental rejuvenation studies have demonstrated that young blood can reverse many of these hallmarks. Heterochronic parabiosis and young plasma infusion rejuvenate stem cell activity^7^, improve metabolic function^8^ and neurogenic rejuvenation^9,10^, restore mitochondrial metabolism^11^, and reset epigenetic age across multiple organs^12,13^. Small extracellular vesicles (sEVs) and specific plasma fractions mediate these effects, supporting the idea that blood-borne molecules act as reprogramming cues^14^. Conversely, the removal of aging factors through plasma dilution or therapeutic plasma exchange promotes systemic rejuvenation without the need for young donor plasma, offering a complementary translational pathway.

Together, these findings position the circulatory system as both a biological archive of aging and a therapeutic interface for its modulation. By decoding the molecular determinants that govern blood-mediated aging, it may become possible to design precision interventions that extend healthspan, delay age-related pathologies, and ultimately reframe aging as a modifiable aspect of human biology.

### Changes in blood components with aging

#### Changes in plasma proteins with aging

Plasma proteins, secreted by various organs and cells circulating throughout the body, reflect the physiological state and pathological changes of individual organs. Owing to these characteristics, plasma proteins have become key non-invasive indicators in aging research, and their functional roles are increasingly being clarified by high-resolution proteomics and large-scale population studies. In particular, plasma proteins serve as more than age-dependent indicators. They are also being explored as potential tools for estimating biological age, monitoring organ-specific aging patterns, and assessing disease risk (Table 1).

**Table 1:** Age-related alterations in plasma proteins.

	Species	Cohort/models	Analysis/tools	Outcomes	Ref.
**Plasma** **proteins**	Human	UK (UK Biobank), China (China Kadoorie Biobank), Finland (FinnGen) 51,408 individuals	2897 (Olink)	Proteomic aging clock. Age prediction accuracy (*r* = 0.92–0.94). ProtAgeGap → Mortality rate, strong association with 18 chronic disease risks	^3^
	Human	5 cohorts, USA and Europe (Knight-ADRC, Covance, LonGenity, SAMS, Stanford-ADRC) 5676 individuals	4778 (Somascan)	856 organ-enriched. Organ-specific age prediction models. Prediction of Alzheimer disease (AD) progression (similar to pTau181 levels)	^16^
	Human	Large-scale proteome (more than based on 4000 individuals) +Human Protein Atlas	2925 (Somascan)	Tissue-specific aging marker: liver (HP, MBL2), lung (SFTPC), pituitary gland (PRL, POMC), pancreas (GCG, CPA2), muscle (MYBPC1), parathyroid gland (PTH), immune cells (MPO, ACP5)Tissue-specific aging markers: liver (haptoglobin, HP; mannose-binding lectin 2, MBL2), lung (surfactant protein C, SFTPC), pituitary gland (prolactin, PRL; proopiomelanocortin, POMC), pancreas (glucagon, GCG; carboxypeptidase A2, CPA2), muscle (myosin-binding protein C, slow type, MYBPC1), parathyroid gland (parathyroid hormone, PTH), and immune cells (myeloperoxidase, MPO; acid phosphatase 5, tartrate resistant, ACP5).	^15^
	Human	UK (UK Biobank) 44,498 individuals 17 years tracking	2916 (Olink)	Brain aging → 8.3-fold increased mortality risk. Young brain/immune system → associated longevity	^17^
	Human	23 cohorts (USA, Europe, and Asia) 18,645 individuals	7000 (Somascan)	Disease-specific protein apolipoprotein E ε4. Common plasma signatures in AD, PD, ALS, and frontotemporal dementia. Organ aging patterns. The world’s largest open-access neurodegenerative proteomic data setDisease-specific protein apolipoprotein E ε4 (APOE ε4). Common plasma signatures in Alzheimer’s disease (AD), Parkinson’s disease (PD), amyotrophic lateral sclerosis (ALS), and frontotemporal dementia (FTD). Organ aging patterns. The world’s largest open-access neurodegenerative proteomic dataset.	^18^
	Human	China (Guangzhou Nutrition and Health Study) 3796 individuals 9 years tracking	438 (MS)	86 proteins associated with aging. PHAS (proteomic healthy aging score) based on 22 aging-associated proteins. → Prediction of cardiovascular and metabolic diseases	^19^

A representative achievement is the development of plasma protein-based aging clocks based on large-scale cohorts. Argentieri et al.^3^ analyzed approximately 2900 plasma proteins from more than 45,000 participants in the UK Biobank. This research presented a ‘proteomic aging clock’ composed of 204 proteins, which not only showed high accuracy in age prediction but also simultaneously predicted the onset and mortality risk of 18 major chronic diseases, including cardiovascular disease, kidney disease, liver disease, neurodegenerative diseases, and cancer. Notably, the difference between proteomic age and chronological age was strongly linked to established aging markers such as telomere length, frailty index, and cognitive decline, demonstrating that the plasma protein clock is an effective tool for reflecting clinical functional decline.

Another advancement in plasma protein interpretation emerged from approaches focusing on tissue specificity. Okada^15^ integrated and analyzed the Human Protein Atlas and large-scale proteomic data, thereby identifying 17 tissue-specific and cell-specific aging-related proteins. This analysis confirmed that these proteins, originating from various distinct tissues and cell types, reflect the aging status of specific organs and cell populations. This approach goes beyond viewing plasma proteins as mere systemic indicators, highlighting their potential to track the aging status of individual tissues by using the tissue specificity. Subsequently, Oh et al.^16^ reported organ-specific aging rates in humans based on plasma protein features specific to 11 major organs and validated the potential for health and disease prediction. Approximately 20% of the population showed significantly accelerated aging in only specific organs, whereas about 1.7% exhibited multi-organ aging. Individuals with accelerated organ aging exhibited a 20–50% higher mortality risk. For example, accelerated heart aging increased the risk of heart failure by 2.5-fold, and aging of the brain and vasculature independently predicted Alzheimer disease (AD) progression. Additionally, kidney aging was closely related to metabolic diseases such as hypertension and diabetes, and muscle aging was associated with gait impairment. This indicates that aging does not occur uniformly throughout the body but may follow distinct trajectories in different organs. More recently reported research elucidated the association among organ-specific aging, healthspan, and longevity through plasma protein-based analysis. Specifically, aging of the brain and the immune system were identified as key factors determining longevity and healthspan. Individuals with younger brains showed more than 70% lower risk of developing AD, and groups with suppressed immune system aging exhibited mortality rates reduced by more than half. These findings suggest that the brain and immune system are central axis determining systemic health and longevity among organ-specific aging trajectories, which describe the distinct temporal and molecular patterns through which individual organs undergo functional decline and remodeling during aging. This indicates that future anti-aging interventions should focus on maintaining and rejuvenating these systems^17^.

Plasma proteins also have a crucial role in neurodegenerative disease research. A large-scale analysis by the Global Neurodegeneration Proteomics Consortium (GNPC), published by Imam et al.^18^, secured approximately 250 million protein measurements from more than 35,000 blood and cerebrospinal fluid samples. This study identified proteins specific to neurodegenerative diseases such as AD, Parkinson disease (PD), amyotrophic lateral sclerosis, and frontotemporal dementia, while confirming that apolipoprotein E ε4 carrier status showed a consistent pattern of protein changes across these diseases. Furthermore, growth differentiation factor 15 (GDF15), a protein closely linked to aging, metabolic stress, and inflammatory responses, was presented as a marker reflecting disease progression and multi-organ aging processes, as it is elevated in the plasma of patients with AD. Glial fibrillary acidic protein, a representative protein indicating astrocyte reactivity and neuroinflammation, was markedly associated with clinical severity in patients with AD and various other dementias, with changes observed in apolipoprotein E ε4 carriers, establishing it as an independent biomarker of neurodegenerative processes transcending specific diseases. Kallikrein-related peptidase 6, an enzyme involved in proteolysis and neuroprotective mechanisms, showed altered expression in both PD and AD, reflecting neurodegenerative pathways. Brevican, a key protein maintaining synaptic stability and extracellular matrix integrity, proposed as a molecular indicator reflecting neural network collapse and synaptic loss, as it was decreased in patients with AD and frontotemporal dementia. This suggests that plasma proteins can contribute not only to disease diagnosis, prognosis, and prediction but also to the discovery of therapeutic targets.

Research attempting to clarify the temporal changes of aging proteins has also been conducted. Tang et al.^19^ tracked 3796 middle-aged and older adults in the Guangzhou region of China for 9 years, analyzing 7565 serum samples at three time points. As a result, 86 aging-related proteins were identified, and a ‘proteomic healthy aging score (PHAS)’ composed of 22 of these proteins was created, which strongly predicted the long-term risk of cardiovascular and metabolic disease development. A notable finding was the close association of PHAS with specific gut microbial species, demonstrating that plasma protein changes are not merely intrinsic molecular processes but are also intimately connected to environmental and lifestyle factors. The longitudinal multi-omics study at Stanford University also presented distinct ‘ageotypes’ for different people, categorizing them into immune/inflammatory, metabolic, hepatic, and renal types. This supports the notion that plasma protein-based analysis can be utilized for personalized aging tracking and management strategies^20^. Meanwhile, plasma proteins not only function as indicators reflecting aging but also as factors that directly act on the aging process^21^. This aspect is discussed in more detail in the review by Kang and Yang^22^. This shows that plasma protein research can be extended beyond simple diagnostic indicators to strategies for the development of anti-aging therapeutics.

In summary, the results mentioned earlier suggest that plasma proteins hold multilayered significance in aging research. Large-scale population-based clocks can precisely calculate an individual’s biological age, and organ-specific protein analysis provides crucial clues for interpreting non-uniform aging processes. They also contribute to the early prediction and target discovery for various age-related diseases, including neurodegenerative disorders. Most importantly, the tracking of plasma protein changes on an individual level is laying the foundation for designing personalized health management and disease prevention strategies. This clearly demonstrates the position of plasma protein research in precision medicine aimed at extending healthspan.

#### Changes in plasma metabolites with aging

Plasma metabolites are a collection of molecules that most closely reflect the physiological changes occurring in cells and tissues, positioning them as an important resource for elucidating the molecular characteristics that manifest during the aging process. Advances in high-resolution mass spectrometry and NMR-based technologies have enabled the tracking of plasma metabolite changes in large-scale cohorts, thereby progressively revealing specific metabolomic indicators of aging (Table 2).

**Table 2:** Age-related alterations in plasma metabolites.

	Species	Cohort/models	Analysis/tools	Outcomes	Ref.
**Plasma** **metabolites**	Human	Japan, Korea 30 individuals (young aged 29 ± 4 years; old aged 81 ± 7 years)	126 (LC-MS)	14 aging-associated metabolites. Decrease in antioxidants and energy metabolism-related metabolites with age. Increase in metabolites linked to kidney/liver function decline. Red blood cell-specific metabolites as novel aging biomarkers	^23^
	Human	Canada 236 individuals	186 (LC-MS/MS)	Differences in metabolites and ratios related to age and sex (especially sphingolipids and phosphatidylcholines accounted for 80%)	^25^
	Human	USA (Wisconsin WRAP cohort) 1212 individuals	1097 (UHPLC-MS/MS)	623 metabolites associated with aging. Sphingolipid; increase in females, decrease in males. Amino acids mainly increase. Sex-specific metabolite trajectories	^24^
	Human	USA (NHS, NHSII, HPFS13) 11,643 individuals; Spain (PREDIMED) 1878 individuals; 14.5–22.6 years of tracking	243 (NMR spectroscopy)	107 mortality and longevity-associated metabolites. Specific nucleosides and ceramides ↑→ Mortality risk ↑ L-serine and polyunsaturated lipids ↑→ longevity Prediction of mortality risk using a multimetabolite score	^26^
	Human	UK (UK Biobank) 250,341 individuals	325 (NMR spectroscopy)	54 key biomarkers using LaSSO-Cox. Mortality/frailty prediction markers (GlycA increase → mortality risk ↑ PUFA-related lipids → mortality risk ↓). Metabolite-based aging score	^28^

Initial studies reported a distinct change in the concentration of individual metabolites with age. Analysis of red blood cells and plasma confirmed that antioxidant and energy metabolism-related molecules such as NAD⁺, carnosine, and ophthalmic acid decrease with aging, whereas molecules such as dimethyl-guanosine and citrulline increase^23^. This reflects the metabolic imbalance associated with aging, such as the accumulation of oxidative stress and reduced efficiency of the urea cycle. Subsequent large-scale cohort studies revealed that sphingolipid and phospholipid metabolism changes in an age-dependent manner and follows different trajectories in males and females. Longitudinal studies are significant because they allow the tracking of changes in metabolic trajectories over time. Research conducted within the Wisconsin Registry for Alzheimer's Prevention (WRAP) cohort showed that more than half (56.8%) of the 1097 plasma metabolites were associated with age, and a considerable number showed different trajectories according to sex. In particular, most steroid lipids decreased with age, but sphingolipids increased in women and decreased in men, highlighting the complexity and diversity of the aging process^24,25^.

Large-scale prospective analyses emphasized that plasma metabolites act as powerful predictors of mortality and longevity. A major cohort study involving a total of 13,512 individuals in the USA and Spain found that nucleic acid metabolites such as *N*^2^,*N*^2^-dimethylguanosine, pseudouridine, and *N*^1^-acetylspermidine were associated with increased mortality, whereas L-serine and highly unsaturated lipids were positively correlated with longevity^26^. Furthermore, a 35-year follow-up study based on the Swedish AMORIS cohort showed that the blood metabolite profile after age 65 could predict survival to age 100 or more, with lower levels of glucose, uric acid, and creatinine and higher levels of total cholesterol and iron being associated with longevity^27^. More recently, metabolite-based aging clocks have garnered attention as a tool to precisely estimate biological age. A study using UK Biobank data analyzed 325 NMR blood metabolite biomarkers from 250,000 individuals to construct a metabolite-based aging score. This score showed performance superior to existing aging indices in short-term mortality prediction, indicating its utility as a tool for assessing individual aging pace and determining the potential for anti-aging interventions. Longitudinal tracking also revealed that inflammation-related indicators such as glycoprotein acetylation (GlycA) are closely associated with accelerated aging^28^.

However, technical limitations exist for comparison between studies. Differences between plasma and serum, anticoagulant processing methods, baseline metabolite differences by sex and age, and the lack of consideration for lifestyle and environmental factors can act as significant confounding variables in the interpretation of research results. Therefore, standardized pre-analytical procedures, sophisticated statistical modeling accounting for sex and age, repeated measurements and longitudinal studies, and integrated analysis using multi-omics are essential. Through these steps, we can move beyond the significance of individual metabolites to understand the entire network that mediates aging and disease risk^29^.

The accumulated evidence to date demonstrates that plasma metabolites accurately reflect the aging process and are promising indicators that can be used for predicting mortality and longevity and, furthermore, for the calculation of biological age. Specifically, nucleic acid metabolites, amino acids, sphingolipids, and phospholipids consistently show aging-related patterns, and interindividual and sex-based variations are also evident. These findings suggest that metabolite-based aging clocks can develop into crucial tools for assessing the efficacy of anti-aging interventions. Personalized aging monitoring using plasma metabolites offers a core possibility in the era of precision medicine and is expected to contribute to the development of strategies for extending healthspan in the future.

#### Changes in blood immune cells with aging

Aging fundamentally reorganizes the composition of immune cells in the blood, and these changes lead to a weakening of immune defenses and an increased vulnerability to disease. Studies using single-cell transcriptome analysis have confirmed that naive T cells and B cells, which constitute a high proportion in young adults, sharply decline with aging, whereas dysfunctional T cells are accumulated (Table 3). Simultaneously, the increase of late-stage NK cells, inflammatory monocytes, age-associated B cells, and functionally impaired dendritic cells leads to the formation of a state of chronic low-grade inflammation, known as inflammaging, which elevates the risk of infection, autoimmune diseases, and metabolic disorders^6^.

**Table 3:** Age-related alterations in blood immune cells.

	Species	Cohort/models	Analysis/tools	Outcomes	Ref.
**Blood immune cells**	Human	Japan 7 supercentenarians (age ≥110) versus 5 younger controls	Peripheral blood mononuclear cells (PBMCs) (scRNA-seq and scTCR-seq)	Clonal expansion of cytotoxic CD4 T cells and preserved IFNγ, TNF-α secretion capacity in supercentenarians → unique immune adaptation contributing to longevity	^37^
	Human	China 188 individuals	Immune cell subsets (scRNA-seq, CyTOF, scATAC-seq, scTCR/BCR-seq)	Functional bias in T cells (effector/exhausted increase), accumulation of proinflammatory monocytes and age-associated B and NK cells	^6^
	Human	US and multinational countries 1214 individuals	IgG Fc N-glycan structure (capillary electrophoresis, MS)	Increased pro-inflammatory agalactosylation, a decreased anti-inflammatory sialylation in HIV infection. → Accelerated biological aging and higher comorbidity risk	^33^
	Mouse	Aged mice with depletion of myeloid-biased hematopoietic stem cells	Bone marrow HSC, blood, plasma, and spleen (myeloid-biased HSC depletion, flow cytometry, bulk RNA-seq)	Identification of surface markers (CD150, CD41, CD62p, NEO1). Distinguishing my-HSCs and balanced-HSCs (bal-HSCs)Identification of surface markers (cluster of differentiation 150, CD150; cluster of differentiation 41, CD41; cluster of differentiation 62P, CD62P; neogenin-1, NEO1) and distinguishing myeloid-biased hematopoietic stem cells (my-HSCs) and balanced hematopoietic stem cells (bal-HSCs).	^35^
	Mouse	9-month AD (APP/PS1 TG) mice, 2-month young BMT mice 9-month-old Alzheimer’s disease (AD) mice (amyloid precursor protein/presenilin-1 transgenic, APP/PS1 TG) and 2-month-old young bone marrow transplantation (BMT) mice.	PBMCs (scRNA-seq, MS, flow cytometry)	Young BMT → Reduction of senescence-associated secretory phenotype (SASP)proteins, amelioration of Aβ plaques and neuroinflammation, and improvement of cognitive function	^36^
	Mouse	Mice with T cell-specific mitochondrial dysfunction (Tfam^fl/fl^ ;Cd4Cre)	Cytokine alterations (flow cytometry, metabolic assays)	Loss of mitochondrial dysfunction in T cells → cytokine storm → accelerated aging, multimorbidity, and reduced lifespan	^32^
	Mouse	Mice with immune cells accumulating DNA damage (Vav-iCre; Ercc1^fl/−^)	Immune cells (flow cytometry, CyTOF, ELISA)	Immune cell aging → Systemic organ aging. Young immune cell transplantation → organ rejuvenation Suppression of immune aging with rapamycin	^30^

The fact that immune system aging causes organ aging has also been demonstrated. Yousefzadeh et al.^30^ constructed a mouse model in which early aging of the immune system was induced in hematopoietic cells by selectively deleting the DNA repair protein ERCC excision repair 1 (Ercc1). Although this model was initially healthy in its youth, it subsequently showed the loss of specific immune cell populations and premature immune aging, mirroring the changes observed in immune cells in naturally aged individuals. Importantly, this premature aging of the immune system induced widespread cellular senescence in extra-immune organs, with significantly increased indicators of DNA damage, oxidative stress, and cellular senescence in non-lymphoid organs such as the liver, kidney, lung, and muscle. Furthermore, when spleen cells from aged mice or immune aging models were transplanted into young recipients, secondary cellular senescence and tissue damage were induced in various organs of the recipient. Conversely, injecting young immune cells alleviated systemic aging indicators in aged mice. This experimentally demonstrated that the immune system, beyond its mere defense function, directly influences the aging rate and tissue homeostasis of systemic organs.

At the structural level, thymic atrophy acts as a key mechanism. The progressive involution of the thymus reduces the output of naive T cells, contracts TCR diversity, and increases the peripheral egress of autoreactive T cells, simultaneously causing immunodeficiency and inflammation^31^. Furthermore, mitochondrial dysfunction in T cells systemically accelerates aging, with premature aging and multi-organ abnormalities observed in TFAM-deficient mice^32^.

Significant changes are also observed in antibody levels. The Fc region of IgG reflects the functional status and degree of aging of the immune system through changes in its N-glycan pattern. With age, the pro-inflammatory agalactosylation pattern progressively increases, whereas the anti-inflammatory characteristic of sialylation is decreased. These changes go beyond merely structural modification of the antibody, acting to strengthen the activation of innate immune receptors and the complement system, thereby promoting systemic inflammatory responses. Indeed, several large-scale cohort studies have confirmed the age-dependent changes in IgG N-glycans, which are presented as crucial evidence supporting inflammaging at a molecular level. Notably, the IgG glycosylation pattern shows individual differences even within the same age group, positioning it as a sensitive indicator for estimating biological age and the rate of aging. Although these changes are accelerated in conditions of chronic disease or infection, they are essentially understood as a phenomenon reflecting the inherent remodeling of the immune system in aging itself. Consequently, the IgG N-glycan pattern is establishing itself as a new axis of biomarkers for quantifying aging, alongside previously used indicators such as telomere length or epigenetic clocks^33^. Moreover, recent research has shown that IgG is not merely an indicator reflecting aging but can directly act as a causal factor for aging. Yu et al.^34^ demonstrated that IgG accumulates in adipose tissue during the aging process, leading to impaired metabolic function and fibrosis. In this study, IgG activated macrophages, promoting adipose tissue fibrosis mediated by the transforming growth factor-β/SMAD signaling pathway, which led to insulin resistance and chronic inflammation. Specifically, calorie restriction improved metabolic health by reducing IgG accumulation in adipose tissue, but this effect was abolished when IgG was re-injected externally. More remarkably, inhibiting the FcRn receptor, which is responsible for IgG recycling, prevented IgG accumulation in adipose tissue, consequently restoring metabolic health and extending both lifespan and healthspan. This shows that IgG is not a simple aging marker but an active factor causing metabolic aging, suggesting the potential for new therapeutic strategies that regulate IgG accumulation.

Recent research has suggested that the lineage bias of hematopoietic stem cells (HSCs) with aging fundamentally determines the changes in blood immune cells. In young individuals, balanced HSCs that produce lymphocytes and myeloid cells in equilibrium are dominant, but with aging, myeloid-biased HSCs accumulate, leading to reduced lymphocyte production and an excessive increase in inflammatory myeloid cells. Ross et al.^35^ showed that selectively eliminating myeloid-biased HSCs with an antibody restored the proportion of naive T cells and mature B cells in aged mice, reduced age-associated dysfunctional T and B cells, and the levels of inflammatory cytokines in the blood. Furthermore, this intervention restored antigen-specific CD8^+^ T cell responses after vaccination and enhanced resistance to viral infection to the level of young individuals. Therapeutic strategies directly targeting immune cell aging are also being proposed. A study that rejuvenated peripheral immune cells in aged Alzheimer’s model mice by transplanting young myeloid cells showed mitigation of cerebral amyloid pathology and cognitive decline, suggesting that immune rejuvenation can contribute to the improvement of neurodegenerative diseases^36^. Collectively, these findings strongly support that the rebalancing of HSCs and the rejuvenation of immune cells represent promising therapeutic approaches for mitigating age-related immune dysfunction and neurodegenerative diseases, thereby contributing to healthspan extension. Specifically, the ‘youthfulness’ of the immune system and the brain was confirmed to be closely related to longevity, and the risk of mortality increased sharply when multiple organs aged simultaneously^17^.

In centenarians and supercentenarians, a unique adaptation phenomenon, contrasting with the typical pattern, has been reported. Hashimoto et al.^37^ analyzed peripheral blood mononuclear cells at a single-cell transcriptome level in individuals aged 110 and over, revealing that cytotoxic CD4^+^ T cells constituted an exceptionally high proportion in the blood and underwent massive clonal expansion. These cells, unlike typical helper T cells, partially acquired the CD8^+^ T cell program at the transcriptional level, expressing cytotoxic factors such as perforin and granzyme. This conversion is interpreted as a compensatory mechanism to maintain antiviral and antitumor immune responses even in extreme old age. The cytotoxic CD4^+^ T cells observed in supercentenarians, in particular, showed not just an increase in proportion but also massive expansion originating from a single clone, suggesting functional persistence coupled with long-term immune memory maintenance. Thus, the long-lived population, unlike typical aging, possesses a unique adaptive strategy to maintain relatively strong immune defenses in old age through the reorganization of the immune repertoire.

Taken together, these studies clearly demonstrate that the aging of immune cells in the blood is a multidimensional phenomenon that encompasses not only a simple decline in defense capability but also changes in antibody glycosylation patterns, imbalance in hematopoietic origin, unique adaptive immune features in long-lived individuals, and new anti-aging technologies directly targeting immune cells. Blood, as the most readily accessible biological sample, is positioning itself as a critical window for predicting and intervening in human lifespan and healthspan, and immune rejuvenation strategies hold the potential to provide a revolutionary turning point in future aging research and clinical application.

### Systemic rejuvenation through circulatory modulation

#### Effects of parabiosis

The systemic milieu refers to the composition of circulating factors — including proteins, metabolites, cytokines, and immune mediators — that influence tissue function across multiple organs. Emerging evidence indicates that the systemic milieu exerts a profound influence on the aging process and regenerative capacity of tissues.

Parabiosis, a representative model connecting the circulatory systems of young and old individuals, provided a conceptual turning point by demonstrating that young blood could reverse physiological dysfunction in aged individuals. The concept of parabiosis first emerged in the 1950s and 1960s. Conboy et al.^38^ showed that directly coupling young and old mice restored the regenerative capacity of muscle satellite cells, thus proving that the young circulatory environment induces stem cell activation and tissue rejuvenation.

Subsequent studies focussed on identifying the molecular mediators of this phenomenon. Loffredo et al.^39^ reported that the young blood factor GDF11 reversed age-related cardiac hypertrophy. Villeda et al.^40^ confirmed increased expression of synaptic plasticity-related genes in the hippocampus of aged mice. Further research identified a protein derived from human umbilical cord blood as a potent factor that rejuvenates hippocampal function in aged mice^12^, demonstrating that the rejuvenation effect via parabiosis is not a mere phenomenon but is mediated by the action of specific blood factors. This highlights the central importance of blood factors in the mechanistic understanding of aging and the development of rejuvenation strategies.

The mechanism underlying the rejuvenation effect through parabiosis is gradually being elucidated in various recent studies. Recent studies based on multi-omics analysis more clearly show that parabiosis induces molecular and transcriptomic reprogramming^41^. A long-term parabiosis study showed that even after separation, following 3 months of circulatory connection, the aged mice sustained a reduction in epigenetic age^9,42,43^, transcriptomic reorganization, immune modulation^4,8,44,45^, and improvement in physiological function^7^. This implies that the rejuvenation effect is not a transient response but can lead to long-term systemic reprogramming that may extend lifespan.

Furthermore, a single-cell, multi-organ transcriptome map study demonstrated that parabiosis reorganizes various organ-specific stem cells and their surrounding niche environment to a youthful state, including hematopoietic stem and progenitor cells, muscle stem cells, skin hair follicle stem cells, and neural stem cells. Specifically, aged hematopoietic stem and progenitor cells, after exposure to young blood, reverted to a transcriptome landscape similar to that of young adults. Key transcription factors (such as activating transcription factor 3 and activating transcription factor 4) regulating hematopoiesis and lymphocyte differentiation, along with cytokine signaling, were restored, alleviating immune aging and lymphopoiesis decline. It was also shown that tissue-specific stem cells, vascular endothelial cells, macrophages, and microglia, acting as niche cells, were co-reprogrammed, underpinning systemic rejuvenation^7,41^.

Additionally, a single-cell transcriptome study revealed that young blood revitalizes systemic metabolic pathways, including the restoration of mitochondrial electron transport chain gene expression, at the cellular level across 20 organs^46^. The fact that aging universally involves a reduction in transcriptome expression and that young blood can reverse this suggests that aging is a phenomenon largely regulated by the circulatory environment.

Organ-specific single-cell transcriptome analysis in the brain confirmed that parabiosis reverses aging transcriptome signatures in various cell types of aged individuals, with remarkable reprogramming observed particularly in cerebral vascular endothelial cells^47^. In the retina, exposure to young blood inhibited inflammation and cellular senescence and restored visual function by activating the AdipoR1–AMPK–mitochondrial pathway^48^. In the liver and heart, parabiosis restored metabolic function and inhibited fibrosis, with vascular endothelial cells identified as a core cell population that most sensitively mediates these changes^11^. Conversely, the negative effects of old blood on young individuals are also apparent. Kiss et al.^49^ observed that young mice exposed to the blood of aged mice through heterochronic parabiosis exhibited accelerated vascular smooth muscle remodeling and activation of aging transcriptomes.

Building on the concept that circulating factors influence aging, Jeon et al.^50^ showed that heterochronic blood exchange can propagate aging phenotypes from old to young mice. Jeon et al. reported that catheters were inserted into the jugular vein and carotid artery of young and aged mice to enable blood exchange between the two. The result confirmed that a widespread aging response was induced in the young mice, with increased expression of p16INK4a, accumulation of SA-β-gal-positive cells, and elevated DNA damage markers such as γH2AX in major organs such as the liver, kidney, and skeletal muscle. Functionally, the young mice showed a significant decrease in muscle strength and endurance, demonstrating that aging factors in the blood can rapidly induce systemic functional decline^50^. This result proves that aging factors can be systemically propagated through body fluids and, simultaneously, that single blood exchange can be a powerful model for the transfer of aging.

**Fig. 1:**
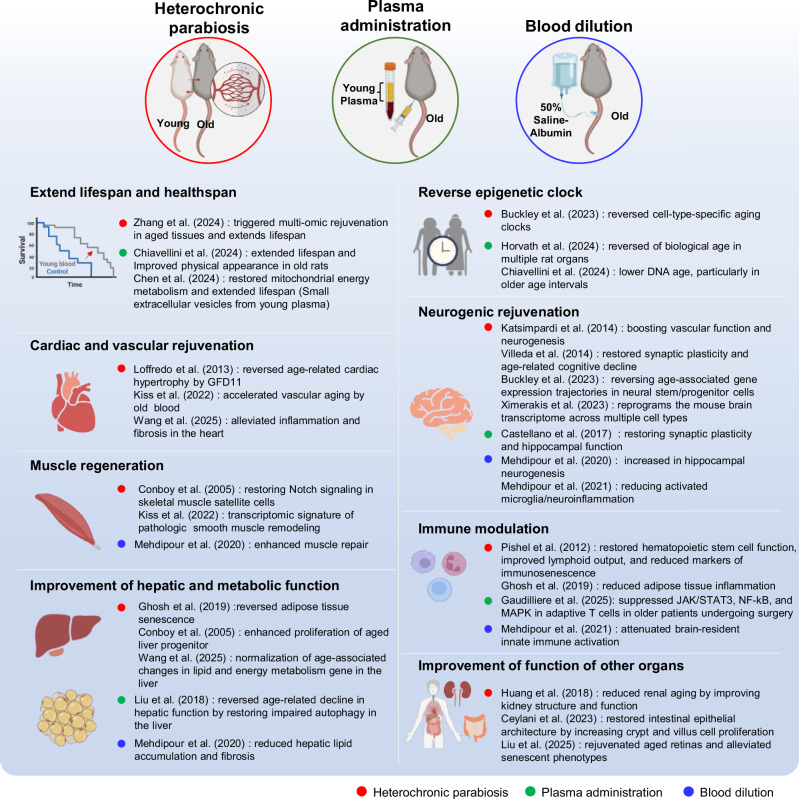
Systemic interventions that modulate aging and promote multi-organ rejuvenation. Heterochronic parabiosis, young plasma administration, and blood dilution improve aging phenotypes across multiple organs. These interventions extend lifespan and healthspan, reset epigenetic aging markers, enhance neural and cognitive function, promote cardiac, muscle, and liver rejuvenation, modulate immune responses, and improve other organ systems. The figure was created using BioRender.com.

Accumulating evidence from parabiosis studies demonstrates that young blood can induce long-lasting, multi-organ rejuvenation by reprogramming molecular and cellular aging signatures. These discoveries mark a conceptual breakthrough in understanding aging as a reversible, systemically regulated process. However, ethical and clinical limitations preclude direct application in humans, underscoring the need to pinpoint specific rejuvenating factors and develop targeted therapeutics. Together, these insights establish parabiosis as a foundational model for advancing translational strategies to promote healthy aging and extend healthspan (Fig. 1).

#### Effects of young plasma administration

Recent advances in aging biology have highlighted young plasma as a potent modulator of systemic rejuvenation. As the acellular fraction of blood, plasma contains numerous bioactive molecules that can influence interorgan communication. Administration of young plasma has been shown to restore youthful molecular and functional states in aged tissues, supporting the idea that soluble circulating factors have a pivotal role in modulating aging. Unlike parabiosis, plasma infusion isolates the effect of humoral factors, offering a safer and more targeted strategy for translating rejuvenation research toward clinical application (Fig. 1).

Building upon earlier observations that young blood can modulate systemic aging, Chiavellini et al.^42^ provided compelling experimental evidence that plasma infusion alone can reprogram epigenetic aging and improve organismal healthspan. Repeated administration of young plasma to aged female rats reversed the blood DNA methylation clock to a younger state, extended average lifespan by approximately 2.2 months, and significantly alleviated external signs of aging, such as maintaining coat luster and activity. Specifically, the epigenetic age in the 27-to-31.5-month interval was significantly lower compared with the control group, and whole-genome methylation analysis showed differential changes in about 1.6% of all CpG sites. Among these, the promoters of genes related to insulin-like growth factor signaling were hypomethylated, suggesting the activation of metabolic and growth pathways, whereas the promoters of cytokine and chemokine-related genes were hypermethylated, reflecting the suppression of inflammatory responses. These changes were also evident in cluster analysis, in which the young plasma group formed an epigenetic profile distinct from the control group. The fact that some long-lived individuals exhibited a pattern similar to the experimental group verified that young plasma administration recapitulates epigenetic characteristics associated with aging suppression and longevity^42^. This epigenetic rejuvenation is interpreted as important evidence demonstrating that aging is not merely the accumulation of cellular damage but a reversible process.

Numerous organ-specific effects have also been reported. In the liver, young plasma restored the autophagic pathway, promoting the clearance of damaged proteins and organelles, thereby alleviating fat accumulation and fibrosis, improving the reduced regenerative capacity, and correcting overall liver dysfunction^51^. In the kidney, the degeneration and fibrosis of tubular epithelial cells that increase with aging were significantly reduced in the young blood environment; the expression of aging indicator proteins p16 and SA-β-gal decreased, whereas autophagic factors such as autophagy protein 5 and microtubule-associated proteins 1 light chain 3B-II increased, and p62 decreased, promoting the degradation of damaged proteins^52^. Furthermore, apoptosis was significantly reduced in TUNEL analysis, and inflammatory markers IL-1β and IL-6 were suppressed, with pNF-κB activity being restored, leading to a marked improvement in tissue homeostasis^52^. In the intestine, young plasma administration inhibited the expression of inflammatory mediator tumor necrosis factor alpha (TNF-α) and cyclooxygenase (COX-2), increased mucosal cell density, and mitigated structural disorganization, resulting in a restoration of tissue structure to a youthful level^53^. Additionally, in the brain, transcriptome analysis of the hippocampus showed restored expression of genes related to synaptic plasticity, increased immediate early genes such as Egr1 and c-Fos and CREB phosphorylation, which led to a higher density of synaptic spines, and improved long-term potentiation. These molecular and structural changes resulted in the recovery of fear conditioning and spatial learning abilities, proving that young blood can reverse cognitive decline^40^. Thus, accumulated organ-specific studies clearly show that young plasma directly alleviates pathological changes and restores tissue-level homeostasis in major organs, including the liver, kidney, intestine, and brain.

A recently reported study revealed that sEVs isolated from the plasma of young mice significantly improved physiological aging indicators in older mice^14^. Administered via repeated intravenous injection, young sEVs extended the lifespan of old mice by ~12%, ameliorated external aging markers such as hair loss, and remarkably lowered the systemic frailty index, making their biological age appear younger than their chronological age. This effect was confirmed to be primarily due to microRNAs within the sEVs inducing PGC-1α expression, thereby restoring mitochondrial energy metabolism^14^. This demonstrates that plasma extracellular vesicles are not mere by-products but active mediators that modulate the systemic aging process.

#### Effects of aged plasma dilution

As aging progresses, structural and functional abnormalities accumulate in circulating blood factors, manifesting as various forms such as inflammatory agents, senescence-associated proteins, and lipotoxins. These changes accelerate systemic aging phenomena such as impaired tissue regeneration, chronic inflammation, and neurodegeneration.

A recently highlighted strategy involves inducing systemic rejuvenation by reducing the excessive concentration of these aging factors and by resetting signaling networks through plasma dilution and therapeutic plasma exchange. This new approach, which focuses on the removal of aging factors rather than the supplementation of young factors, presents a novel perspective in aging research (Fig. 1).

Mehdipour et al.^54^ provided evidence supporting this paradigm shift. Neutral blood exchange, which involved replacing the plasma of aged mice with saline and albumin, promoted muscle regeneration, reduced hepatic fat accumulation and fibrosis, and increased neurogenesis in the hippocampus. The fact that rejuvenation effects occurred without simply supplementing young blood factors validates the concept that the accumulation of aging factors is central to tissue functional decline^54^. Subsequently, an animal study showed that blood dilution improved cognitive function in aged mice and alleviated neuroinflammation by suppressing the excessive activation of microglia. Proteomic analysis supported the cognitive improvement effect, which was not a mere behavioral phenomenon, by showing the restoration of suppressed neuroprotective and neurogenic factors after dilution^4^.

The strategy is now also expanding into clinical application. A small-scale clinical study showed that the proteomic composition of individuals who underwent repeated plasma dilution shifted overall to a younger pattern; indicators of DNA damage and cellular senescence decreased; and the balance of immune cell composition was restored. Importantly, core signaling pathways such as JAK–STAT, MAPK, NF-κB, and Toll-like receptor were normalized, suggesting that biological age can actually be reversed^55^. This result emphasizes that blood dilution does not merely affect blood composition but has the effect of resetting systemic signaling networks. However, as this study was limited to a small clinical scale, validation through larger-scale, randomized controlled clinical trials and long-term follow-up studies is essential.

Kang et al.^56^ reported that plasma exchange in patients with AD could remove persistent organic pollutants. A detailed analysis of the Alzheimer’s Management by Albumin Replacement (AMBAR) clinical trial showed some improvement in cognitive function, neuropsychiatric symptoms, and quality-of-life indices. Specifically, patients with mild Alzheimer experienced improvement in verbal fluency and processing speed, whereas moderate patients showed better short-term memory^57^. Neuroimaging studies confirmed protective effects at the structural and functional levels, such as the slowing of atrophy in key structures such as the hippocampus and a delayed rate of brain metabolic decline^58^. This demonstrates that plasma dilution (albumin–saline exchange) can improve not only the clinical symptoms of patients with AD but also the brain’s anatomical and metabolic indicators.

### Clinical studies on plasma therapy and aged plasma dilution

The translation of young plasma research from preclinical models to human trials is underway, reflecting its therapeutic potential while also raising important regulatory considerations. Early clinical research based on young plasma is best exemplified by the application of the plasma-derived fraction GRF6019, developed by Alkahest, to patients with AD. Hannestad et al.^59^ reported the development of GRF6019, a refined plasma protein fraction manufactured from large-scale pooled plasma from young, healthy donors. Repeated intravenous infusion of GRF6019 in patients with mild-to-moderate AD was confirmed to be safe and well tolerated, with no serious adverse events reported in either dose group (100 ml and 250 ml). Subsequently, a placebo-controlled, double-blind phase 2 clinical trial administered 250 ml intravenous infusions for 5 consecutive days to patients with severe AD with MMSE scores in the 0–10 range. All patients completed the treatment, and no serious adverse events were observed. Adverse events were mostly mild, such as transient blood pressure changes, with an incidence similar to the placebo group.

Although cognitive and functional assessments showed an overall tendency toward stabilization or some improvement in both groups, the limited study size and duration constrained the ability to prove statistically significant efficacy. Nevertheless, these preliminary clinical results demonstrate that plasma fractions derived from young blood can be developed as a safe and repeatable therapeutic strategy for patients with neurodegenerative brain diseases, suggesting the need for future large-scale, long-term studies to elucidate actual cognitive improvement effects^13^. Furthermore, a randomized double-blind clinical trial administered a plasma protein fraction from young donors to older patients undergoing hip and knee replacement surgery, resulting in a significant modulation of postoperative inflammatory and immune responses. Specifically, inflammation-related pathways such as NF-κB, JAK-STAT, and MAPK were suppressed, and significant changes were also observed in single-cell immune analysis^44^. This is important evidence suggesting that components of young plasma can indeed exert rejuvenation effects through immune and inflammation modulation in humans.

Regulatory considerations are also necessary alongside this clinical potential. The cases of some companies commercially offering young plasma transfusions to the older patients were deemed scientifically unsubstantiated, leading to a strong warning from the FDA in an official statement in 2019. The FDA clearly stated that administering plasma from young donors for anti-aging, memory enhancement, or treatment of AD or PD has unproven clinical efficacy and is not an approved therapy. They also warned of serious risks such as allergic reactions, infections, circulatory overload, and lung injury (transfusion-related acute lung injury), emphasizing that such procedures must be conducted only within clinical trials under an investigational new drug protocol^60^. This is viewed as a regulatory mechanism to prevent commercial exploitation when the scientific validation of plasma-based therapies is insufficient.

## Discussion and conclusion

Collectively, the evidence reviewed here supports a conceptual shift in aging biology, positioning the circulation not merely as a passive biomarker of aging but an active determinant shaping systemic decline or rejuvenation. Blood increasingly appears not only as a passive indicator of aging but also as an active determinant shaping systemic decline or rejuvenation. Multi-omics profiling has revealed that circulating proteins, metabolites, and immune cell states encode organ-specific aging patterns and serve as strong predictors of healthspan and disease risk. These insights redefine aging as a systemically regulated process influenced by dynamic interorgan communication, rather than one driven solely by cell-autonomous deterioration.

At the translational frontier, interventions that modulate the circulatory environment — young plasma infusion, sEVs, and plasma dilution — demonstrate that systemic aging can be partially reversed. These approaches restore transcriptomic and metabolic youthfulness, enhance stem-cell and mitochondrial function, reduce inflammatory signaling, and improve cognitive and physical performance in aged animals. Early-stage human studies further show safety, immune recalibration, and potential deceleration of biological aging, emphasizing the clinical promise of circulatory interventions.

Despite this progress, several critical and key challenges remain: identifying causal pro-aging and rejuvenating factors, establishing robust biomarkers of response, and validating efficacy in rigorous randomized trials. As mechanisms become clearer, circulatory modulation may enable precision, mechanism-based strategies to maintain tissue homeostasis, delay age-related diseases, and ultimately extend human healthspan.
